# Mitral Valve Echodensities in a Young-Adult Female with Relapsing Polychondritis, Transiently Positive Lupus Anticoagulant, and Systemic Embolism

**DOI:** 10.1155/2023/5073128

**Published:** 2023-10-07

**Authors:** Michael C. Sauer, Vikram Sharma, Jennifer L. M. Strouse, Ramzi El Accaoui, Christopher J. Benson

**Affiliations:** University of Iowa Hospitals & Clinics, 200 Hawkins Dr, Iowa City, IA 52242, USA

## Abstract

**Background:**

Valvular strands seen on echocardiography carry a wide differential diagnosis and may not always have a clear etiology despite taking clinical context into account. The decision of whether to provide anticoagulation for these lesions can be challenging. *Case Presentation*. A young adult female with an extensive rheumatologic history involving relapsing polychondritis and positive lupus anticoagulant presents to the emergency department with a discolored and painful right toe, as well as right auricular pain and swelling. Initial work-up revealed a possible splenic infarct, vasculitis of the right lower extremity, and mitral valve echodensities on echocardiography, without evidence of infective endocarditis. Due to concern that nonbacterial thrombotic endocarditis may be the cause of the patient's thromboembolic event, her valvular lesions were treated with low molecular weight heparin while awaiting serial imaging. When follow-up echocardiography showed no change in the size of her mitral valve lesions, which would be most consistent with Lambl's excrescences, the care team still faced a decision about which long-term anticoagulation to prescribe. This patient of childbearing age wished to avoid the teratogenicity and long-term monitoring associated with warfarin therapy. Although warfarin was the preferred agent for the patient's rheumatologic comorbidities, she elected to receive enoxaparin therapy for long-term thromboembolism prophylaxis.

**Conclusions:**

Even when accounting for clinical context, valvular lesions seen on echocardiography often have uncertain etiology and may require time and serial imaging to determine which treatment to pursue. When long-term anticoagulation is provided for females of childbearing age, shared decision-making with consideration of the patient's personal priorities and comorbidities is essential.

## 1. Introduction

Native valve lesions can be caused by a wide range of both pathologic and benign abnormalities that may not have an obvious etiology at the time of discovery [1]. Nonbacterial thrombotic endocarditis (NBTE) is one condition that may warrant treatment in patients who have systemic thromboembolism. NBTE is often associated with malignancy, systemic lupus erythematosus, and antiphospholipid syndrome [2–4]. However, equivocal imaging findings may require serial imaging to determine their true etiology and the best long-term treatment. In patients presenting with a rheumatologic history and systemic thromboembolism, warfarin therapy is often the recommended antithrombotic agent [5, 6]. However, patients' personal preferences and life circumstances, including childbearing, may sway some to seek an alternative anticoagulation agent.

## 2. Case Presentation

A young adult female with a history of relapsing polychondritis, positive lupus anticoagulant, and prior blue toe syndrome of the left foot presents to the emergency department with one month of worsening pain and discoloration of the right toe, as well as right auricular pain and swelling. The toe pain was throbbing in character and worsened with elevation of the foot. She was afebrile with a pulse of 105 beats per minute and otherwise exhibited normal vital signs. Physical exam was notable for purple discoloration of the right first toe, pain and swelling of the right ear helix, absence of a heart murmur, and tenderness with palpation of the left lower ribs. Pulse was found by Doppler in the right big toe.

The patient's history includes relapsing polychondritis that had been diagnosed two years prior, followed by a diagnosis of Sweet syndrome several months later when she developed a lower-extremity rash. On two separate occasions more than twelve weeks apart, the patient was found to have a positive lupus anticoagulant and cardiolipin antibodies. However, she was not diagnosed with antiphospholipid syndrome (APS) at that time due to the absence of any thrombotic events. Additional prior immune work-up revealed positive ASCA IgA and low-positive anti-SSB antibodies. She developed left-sided blue toe syndrome one year prior to the current presentation, with subsequent imaging revealing occlusion of the left anterior tibial artery with distal small caliber reconstitution near the ankle. Vascular surgery had recommended against intervention, and the patient did not begin the prescribed aspirin and nifedipine at that time due to an upcoming nasal reconstruction for her relapsing polychondritis.

The patient's rheumatologic history also includes previous episodes of costochondritis, right auricle chondritis, nasal cartilage inflammation leading to saddle nose deformity, inflammatory arthritis of the ankles and knees, neutrophilic dermatosis, and previous history of skin lesions on the lower extremities concerning for pyoderma gangrenosum. She also has a history of aphthous ulcers, although these improved after discontinuation of methotrexate. The patient has never been pregnant. Therapies prior to admission included colchicine every other day and tocilizumab weekly.

### 2.1. Differential Diagnosis

Given the patient's extensive history of rheumatologic phenomena and her immunocompromised status in the setting of her home medications, the patient's presentation required a wide differential diagnosis. The differential included systemic vasculitis, Behcet's disease (given her history of oral ulcers and pustular skin lesions), MAGIC syndrome (mouth and genital ulcers with inflamed cartilage), inflammatory bowel disease, APS, and infectious endocarditis (considering chronic immunosuppression).

### 2.2. Investigations

**Figure 1:**
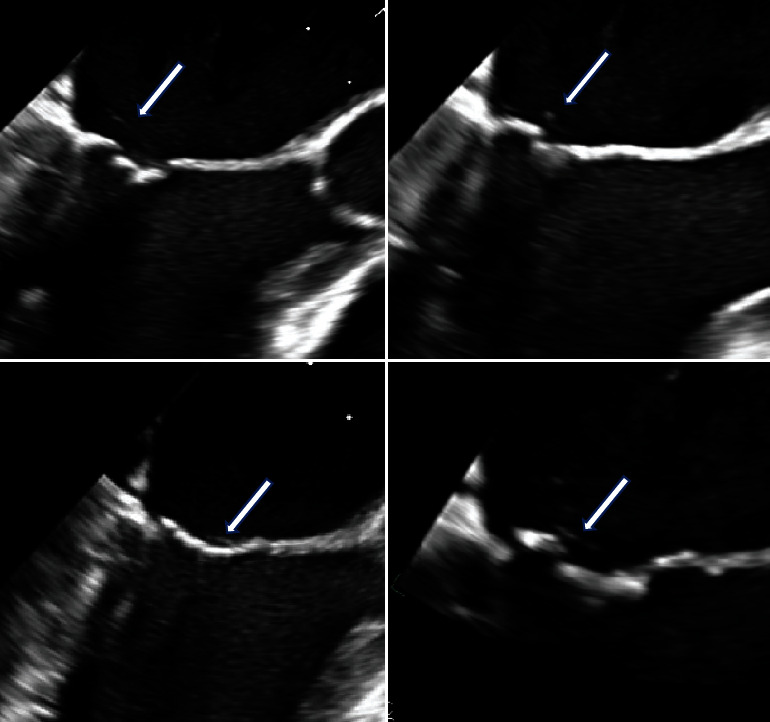
Filamentous echodensities on a mitral valve in a patient with relapsing polychondritis, positive lupus anticoagulant, and signs of systemic embolization.

Laboratory tests were notable for a white blood cell count of 27.3, reactive thrombocytosis with platelets of 442, CRP of 1.0, normal ESR (patient was taking tocilizumab prior to admission), normal ferritin, PTT of 27, and two sets of negative blood cultures. Computed tomography angiography (CTA) of the chest, abdomen, pelvis, and bilateral lower extremities was revealing for wall-thickening and inflammatory changes in the right popliteal artery, bilateral diminutive arteries distal to the popliteal arteries, and a wedge-shaped hypodensity in the superior portion of the spleen that was concerning for an infarct. Hepatitis serologies, syphilis, and tTG-IgA studies were all negative, and immunoglobulin levels were normal. Of note, beta-2 glycoprotein and anticardiolipin antibodies were negative on this occasion. HLA B51, which is associated with Behcet's disease, was negative. Hematology was consulted to assist with work-up for hypercoagulability and felt that APS was unlikely given the lack of definitive evidence of thrombotic occlusions, negative anti-beta-2 glycoprotein, and negative anticardiolipin antibodies at the time of presentation. Vascular surgery was consulted and recommended anticoagulation. Initial transthoracic echocardiogram (TTE) was only significant for possible transpulmonary shunting on agitated saline bubble injection. A subsequent transesophageal echocardiogram (TEE) revealed no evidence of infective endocarditis, but it did show fibrinous strands attached to the posterior mitral valve leaflet (Figure 1), raising the possibility of nonbacterial thrombotic endocarditis (NBTE). The patient did not meet the clinical criteria for infective endocarditis, and multiple blood cultures were negative.

### 2.3. Initial Management

Prior to obtaining TEE, the patient was treated for vasculitis and polychondritis with a pulse dose of methylprednisolone followed by a prednisone taper. A heparin drip was initiated after CT angiography showed the possible splenic infarct, consistent with an embolic event from suspected NBTE. Once infective endocarditis was ruled out with TEE and multiple negative blood cultures, the patient was administered an infliximab infusion. Cyclophosphamide was deferred due to the patient's desire to preserve fertility. She continued her home prescriptions of colchicine and nifedipine while inpatient.

A multidisciplinary discussion was held regarding long-term anticoagulation management. Although NBTE is typically treated with lifelong anticoagulation, preferably enoxaparin, the patient's clinical data was equivocal regarding whether there was concrete evidence of thromboembolic phenomena, or whether her exam and imaging abnormalities were caused by a vasculitis. It was also peculiar that her history of two positive lupus anticoagulants, followed by a negative lupus anticoagulant during admission, gave equivocal evidence of APS. Moreover, the patient did not want to begin warfarin therapy, in part because of a desire to conceive children in the near future. She was ultimately transitioned to enoxaparin 1 mg/kg at discharge with a plan to repeat cardiac imaging in three months for further clinical direction.

### 2.4. Follow-Up

After discharge, vascular surgery started the patient on cilostazol. Rheumatology initiated methotrexate and sildenafil and discontinued nifedipine. Due to her abundant history of rheumatologic phenomena and transiently positive lupus anticoagulant antibodies, the decision was to indefinitely anticoagulate the patient with enoxaparin. Arterial duplex studies showed a maneuver-dependent right popliteal entrapment that did not exist on the left. She was also found to have occluded peroneal, anterior tibial, and posterior tibial arteries with distal reconstitution, as well as occluded left peroneal and anterior tibial arteries. Carotid duplex was normal. The patient had low-positive cardiolipin IgM antibodies on two occasions one month apart, with negative cardiolipin IgG and IgA, and negative beta-2-glycoprotein antibodies.

**Figure 2:**
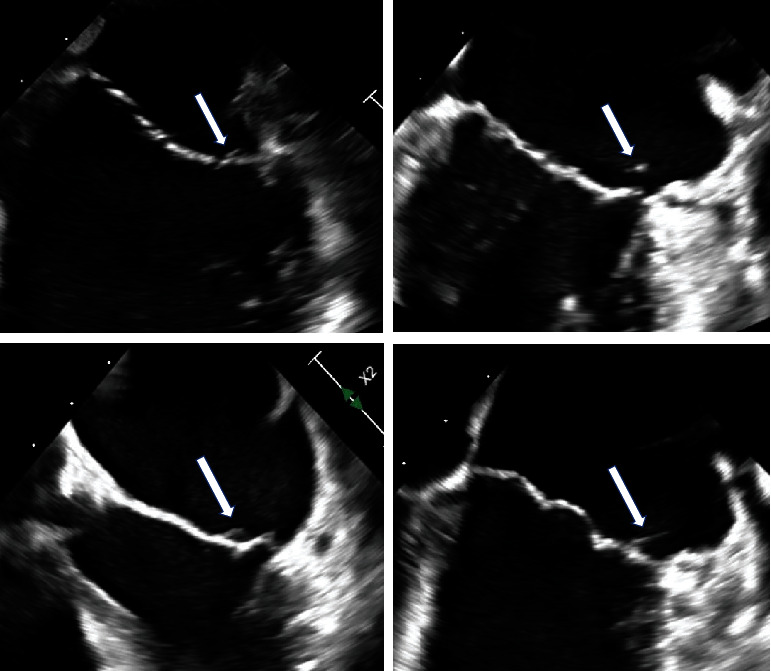
Persistent filamentous echodensities despite systemic anticoagulation and appropriate anti-inflammatory agents.

Repeat TEE 6 months after discharge again showed a filamentous, mobile echodensity about 7 mm in length along the posterior leaflet lateral scallop, unchanged from prior TEE (Figure 2).

The persistence of the fibrin strands on TEE decreased the suspicion for NBTE or a cardioembolic etiology for the patient's clinical presentation. On review, the imaging findings were deemed most likely to be Lambl's excrescences. However, due to the patient's constellation of associated clinical findings, including evidence of splenic infarct and a recurrent rheumatologic history, the decision was made to continue the recommendation of indefinite anticoagulation with enoxaparin.

## 3. Discussion

Echodense, filiform strands on heart valves open the door to a wide differential and require clinical context to determine their etiology and to determine the best treatment. Possible causes of filamentous echodensities on the left-sided heart valves include bacterial and nonbacterial endocarditis, fibroelastomas, myxomas, cardiac thrombi, cardiac tumors, metastases, and Lambl's excrescences [1, 7]. In our patient having negative blood cultures, clinical signs of embolism, and a rheumatologic history, the initial suspicion for NBTE was strong. However, despite treating our patient with anticoagulation for several months, the echodensities persisted. This made NBTE less likely and raised suspicion for Lambl's excrescences being the most likely explanation for the imaging findings.

NBTE is caused by sterile platelet thrombi depositing on the endocardium, most commonly on the mitral or aortic valves [2–4]. It is typically caused by endothelial injury in the setting of a hypercoagulable state, the most common etiologies being malignancy, systemic lupus erythematosus, and APS. The diagnosis is clinical, requiring echocardiographic confirmation of vegetations in the absence of positive blood cultures or other systemic infection [2]. TEE is more sensitive than TTE for visualizing the platelet thrombi. Most patients initially present with noncardiac symptoms, most commonly due to symptoms of thromboembolic events, with cerebrovascular events being the most common [4]. Therapy is targeted toward treating and optimizing the patient's underlying condition in addition to anticoagulation, generally with LMWH [8]. Echocardiography (TTE or TEE) cannot reliably differentiate between bacterial versus nonbacterial endocarditis.

In contrast to NBTE, Lambl's excrescences are acellular, densely hyalinized filamentous processes with a fibroelastic core covered in a single layer of endothelium, located on the valvular cusps [9]. Their pathogenesis is unclear, but most cases remain asymptomatic and are only incidental findings on echocardiography or biopsy. Whether Lambl's excrescences are indeed associated with an increased risk for thromboembolism remains uncertain and controversial in the literature. One cross-sectional study showed that valve strands had a prevalence of 10.6% in patients referred for echocardiography after a systemic embolism compared to only 2.3% of patients without emboli (OR 4.8, *p* = 0.0001) [10]. However, another prospective study followed patients with systemic lupus erythematosus (SLE) for six years and found that Lambl's excrescences had similar prevalence in patients who developed cerebrovascular disease compared to those who did not, even when compared to healthy controls [11]. While NBTE lesions tend to be more rounded and sessile, Lambl's excrescences are filamentous/strand-like echodensities. However, in small-sized lesions, such as in our patient, it may be challenging to differentiate between the two pathologies.

Our patient had other risk factors for a hypercoagulable state, most notably her history of blue toe syndrome, positive lupus anticoagulant, equivocal lab findings for APS, and prior evidence of vasculitis. Given her strong clinical risk for blood clots, it was felt that indefinite anticoagulation would be in the patient's best interest.

This case is of particular interest because there is an accumulation of findings that individually may not significantly increase clinical risk for thromboembolism but, when taken in aggregate, raise high concern for thrombotic risk. This includes the patient's history of a positive lupus anticoagulant, intermittent weakly-positive anticardiolipin antibodies, Lambl's excrescences, a history of relapsing polychondritis, and a history of blue toe syndrome. While vasculitis is a possible etiology for the patient's clinical presentation, this would be less likely to explain her splenic infarct on abdominal CT. Despite this, she presented with imaging findings consistent with vasculitis that may have contributed to—or at least occurred in parallel with—her clinical course.

Another point of interest with this patient is which agent of anticoagulation to choose. In most patients with APS who have a history of venous or arterial thrombosis, the first line of treatment is usually warfarin with the possible addition of low-dose aspirin [6]. Our patient did not have a definite diagnosis of APS due to her intermittently negative antiphospholipid antibodies; moreover, she was personally reluctant to initiate warfarin therapy. One priority that contributed to the patient's reluctance was her desire to bear children in the near future and thus wanting to avoid the teratogenic risks associated with warfarin. In light of the patient's preferences, she was prescribed LMWH.

Warfarin has known teratogenic risks in pregnancy. These risks are highest in the first trimester, with the peak risk occurring during organogenesis from weeks 6 to 12 of gestation [12]. For patients with APS taking warfarin prior to gestation, it is recommended that they switch to a therapeutic dose of LMWH while continuing to take low-dose aspirin [5]. LMWH should then be switched to unfractionated heparin at 36 to 37 weeks gestation and stopped 24 hours prior to delivery. In the absence of significant bleeding, anticoagulation should then be resumed 4 to 6 hours after vaginal delivery or 6 to 12 hours after caesarian delivery.

## 4. Conclusion

Filamentous valvular echodensities can be caused by a large number of etiologies. Even when astutely accounting for clinical clues and context to investigate the etiology of valvular echodensities, the precise diagnosis of these imaging findings may remain unclear and can require time to manifest themselves. In some cases, serial imaging in the outpatient setting can assist with decisions for continued anticoagulation. The decision of whether to anticoagulate in response to these valvular anomalies should be influenced by the patient's comorbidities and clinical history.

Patients with a rheumatologic history and clinical signs of arterial and venous thrombosis may benefit from anticoagulation even in the absence of an absolute diagnosis of APS. The method of anticoagulation should consider the patient's values and goals of care. For example, patients anticipating pregnancy can face difficult decisions regarding the risks and benefits of receiving warfarin in the periconception period.

## Data Availability

All images in this article are available to the public through the publication of this manuscript. No other data was used for this case report.

## Abbreviations

ASCA: Anti-Saccharomyces cerevisiae antibodies
MAGIC syndrome: Mouth and genital ulcers with inflamed cartilage
CRP: C-reactive protein
ESR: Erythrocyte sedimentation rate
PTT: Partial thromboplastin time
CT: Computed tomography
tTG: Tissue transglutaminase
HLA: Human leukocyte antigen
TTE: Transthoracic echocardiogram
TEE: Transesophageal echocardiogram
NBTE: Nonbacterial thrombotic endocarditis
CTA: CT angiography
LMWH: Low molecular weight heparin
APS: Antiphospholipid syndrome.
